# Evaluation of Setting Times of Concrete Using Electro-Mechanical Impedance Sensing Technique

**DOI:** 10.3390/ma16165618

**Published:** 2023-08-14

**Authors:** Jun-Cheol Lee

**Affiliations:** Department of Architecture, Seowon University, Cheongju 28674, Republic of Korea; leejc@seowon.ac.kr

**Keywords:** electro-mechanical impedance, setting time, concrete, piezoelectricity

## Abstract

This study presents a novel approach to assessing the setting time of concrete using the electro-mechanical impedance (EMI) sensing technique. The proposed method involves the continuous monitoring of EMI changes by embedding a piezoelectric (PZT) sensor directly in the concrete. A comparative analysis was conducted with the conventional penetration resistance test, which utilizes mortar samples extracted from the concrete. As a result of the experiment, the time deviation rate of the setting time was more than 10% in the penetration resistance test using the mortar sample extracted from the same concrete, whereas the time deviation rate of the setting time was up to 1.77% in the EMI sensing technique using the same concrete specimen. This highlights the effectiveness and potential of the EMI sensing technique for an improved evaluation of concrete setting time.

## 1. Introduction

Cement is a material that gradually hardens and becomes a solid structure when it comes into contact with water. During the process of cement hydration, a phase transition occurs from a fluid state to a solid state, which is known as setting [1]. The setting time of cement is a significant factor in determining the timing of concrete finishing, prevention of cold joints, initiation of steam curing, and removal of formwork. Therefore, it is very important to clearly identify the setting time of cement [1].

The Vicat needle test [2] and penetration resistance test [3] are most commonly used to evaluate the setting times of cementitious materials. These methods offer advantages such as ease of use, suitability for both field and laboratory environments, and affordability of test equipment. However, the Vicat needle test has limitations in capturing the setting characteristics of mortar or concrete since it is restricted to testing only the cement paste [4,5,6]. The penetration resistance test is a method used for measuring the setting times of mortar. To measure the setting times of concrete using the penetration resistance test, it is necessary to extract mortar from the concrete by sieving. However, it is very difficult to perfectly extract only mortar from concrete by sieving. When extracting mortar through the sieving of concrete, it is possible for material separation to occur, resulting in variations in the water/cement ratio and cement/fine aggregate ratio of the extracted mortar [7,8]. Therefore, the setting times determined by the penetration resistance test using the mortar extracted from concrete do not accurately represent the setting times of concrete.

Recently, as part of non-destructive testing, a method for evaluating the setting of cementitious materials using an electro-mechanical impedance (EMI) sensing technique has been proposed [9,10,11,12,13,14,15,16]. The EMI sensing technique is based on a simple principle. When a Piezoelectric (PZT) sensor is connected to a host structure and subjected to a sinusoidal voltage, it induces vibrations in the coupled area of the structure. These vibrations result from the direct effect of the piezoelectric material. Furthermore, the vibration response of the host structure generates an electrical response, typically in the form of an electric current, in the PZT sensor. This electrical response occurs due to the converse piezoelectric effect, where mechanical deformation induces an electric charge in the piezoelectric material. The EMI can be calculated by analyzing the input and output signals of the PZT sensor [17,18,19].

In previous studies [13,14,15,16], the setting times of cementitious materials were assessed by analyzing the behaviors of the resonant peak and frequency of the EMI of the PZT sensor embedded in the cement paste and the cement mortar. It has been observed that when the PZT sensor is embedded in cementitious material, the EMI signal of the PZT sensor is influenced by the change in stiffness resulting from the hydration of the cementitious material in contact with the PZT sensor. Therefore, the EMI sensing technique using the PZT sensor embedded in concrete is expected to be effective in evaluating the setting times of concrete. This is because it directly measures the changes in stiffness of the concrete, providing valuable insights into the setting process.

In this study, the EMI evolution of the PZT sensor directly embedded in the concrete was continuously monitored to assess the setting time of the concrete. The setting times assessed using the EMI sensing technique were compared with the setting times assessed using the penetration resistance test method, which is a conventional test method. This investigation explored the limitations of the penetration resistance test in accurately evaluating concrete setting times. Moreover, this study comprehensively assessed the suitability of the EMI sensing technique for evaluating the setting of concrete, which is a more intricate mixture than simple cement paste or mortar.

## 2. Experimental Program

### 2.1. Materials

KS L 5201 type I (equivalent to ASTM C 150 [3]) ordinary Portland cement was used for the concrete sample [20]. A typical river sand with a maximum size of 4.75 mm was used as the fine aggregate, while crushed granite stone with a maximum size of 25 mm was used as the coarse aggregate in the concrete mixture. Table 1 shows the physical properties of aggregates used in this study. The concrete was designed with the objective of achieving a compressive strength of 24 MPa at a 28-day age. The concrete mixture had a water-cement ratio (W/C) of 50% and a fine aggregate ratio (S/a) of 37.9%. Table 2 shows the mixture proportion of concrete sample.

### 2.2. Penetration Resistance Test

The penetration resistance test was conducted according to ASTM C 403 [3] in order to compare with the EMI sensing technique for assessing the setting time of the concrete. After the concrete was mixed using a pan mixer, the mortar was extracted from the concrete by sieving the mixture through a 4.75 mm sieve. A cylindrical container with a diameter of 15 cm and a height of 15 cm was placed with the mortar extracted from concrete. Three mortar samples were prepared from the same batch of concrete. Additionally, a mortar sample was prepared by excluding the coarse aggregate from the mixing ratio specified in Table 1, allowing for a comparison with the mortar samples extracted from the concrete.

A penetration needle with an appropriate cross-sectional area was placed in contact with the surface of the mortar, and the needle was penetrated to a depth of 25 mm. The penetration resistance was calculated by dividing the load applied when the needle penetrates to a depth of 25 mm by the cross-sectional area of the needle. The initial measurement of penetration resistance was conducted 1 h after the contact between cement and water. The penetration resistance was measured every hour until the penetration resistance was less than 3.5 MPa, and the penetration resistance was measured every 30 min from the time when the penetration resistance exceeded 3.5 MPa. The time penetration resistance data obtained from the penetration resistance test was subjected to regression analysis using an exponential curve fitting. Using the equation obtained from the regression analysis, the time when the penetration resistance reached 3.5 MPa was defined as the initial setting time, while the time when the penetration resistance reached 27.6 MPa was defined as the final setting time [3].

### 2.3. EMI Measurement

**Figure 1:**
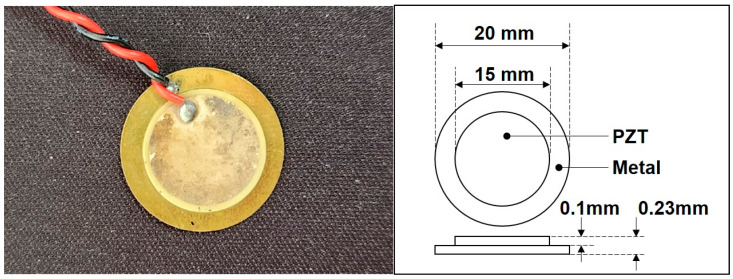
PZT sensor for EMI measurement.

In this study, the EMI evolution of the PZT sensor embedded in concrete samples was monitored to assess the setting time of concrete using the EMI sensing technique. The EMI signal was monitored using a buzzer-type PZT sensor (model CBC2035BA, Daeyoung Electric Co., Ltd., Gyeongsan, Republic of Korea). To prevent any short-circuiting caused by ions dissolved in the fresh concrete, a thin coating of acrylic resin was applied to the surface of the PZT sensor [13]. Figure 1 and Table 3 show the specification of the PZT sensor used in this study.

The concrete samples were cast into a cylindrical container with a diameter of 150 mm and a height of 150 mm, and the PZT sensor was embedded in the center of each container. Three concrete samples were prepared from the same batch of concrete.

**Figure 2:**
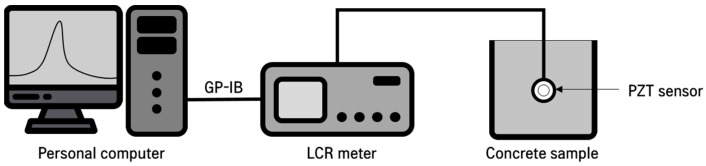
Test setup for measuring EMI of PZT sensor.

The initial measurement of the EMI of the PZT sensor was conducted 1 h after the contact between cement and water, and subsequent measurements were conducted every 10 min for 12 h. The EMI of the PZT sensor was measured using a commercial LCR (Inductance (L), Capacitance (C), and Resistance (R)) meter (Hioki, Dallas, TX, USA, 3235-50 LCR HiTESTER), and all measured data were recorded using a personal computer through a GP-IB interface connected to the LCR meter. The measurement frequency range for the EMI of the PZT sensor was set from 20 kHz to 250 kHz, with a measurement frequency interval of 50 Hz. Figure 2 shows the testing setup for the measurement of the EMI of the PZT sensor.

## 3. Results and Discussion

**Figure 3:**
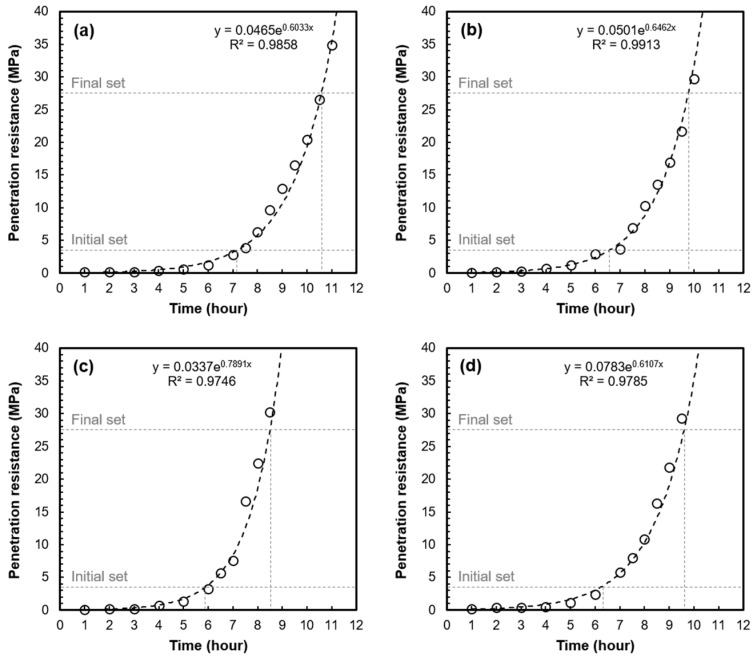
Penetration resistance as function of the age: (**a**) MC1, (**b**) MC2, and (**c**) MC3; mortar samples extracted from the same batch of concrete and (**d**) pure mortar.

Figure 3 shows the penetration resistance of mortar samples extracted from the same batch of concrete as a function of hydration time. Table 4 shows the initial and final setting time of the mortar samples in accordance with ASTM C 403.

As shown in Figure 3 and Table 4, the setting times of the mortar samples extracted from the same concrete batch were different. The difference in the initial and final setting time between MC1 and MC3, which are mortar samples extracted from the same concrete batch, was 77 min and 125 min, respectively. The time gaps between the initial set and final set for MC1 and MC3 were 205 min and 157 min, respectively. It is also worth noting that the initial setting time and final setting time of the mortar extracted from concrete showed deviations of up to 57 min and 66 min, respectively, in comparison to those of the pure mortar. These results indicate that when extracting mortar from concrete through sieving, it is not possible to obtain pure mortar because the water/cement ratio and cement/fine aggregate ratio may differ. The variations in these ratios can lead to differences in setting times of the mortar samples extracted from the concrete.

**Figure 4:**
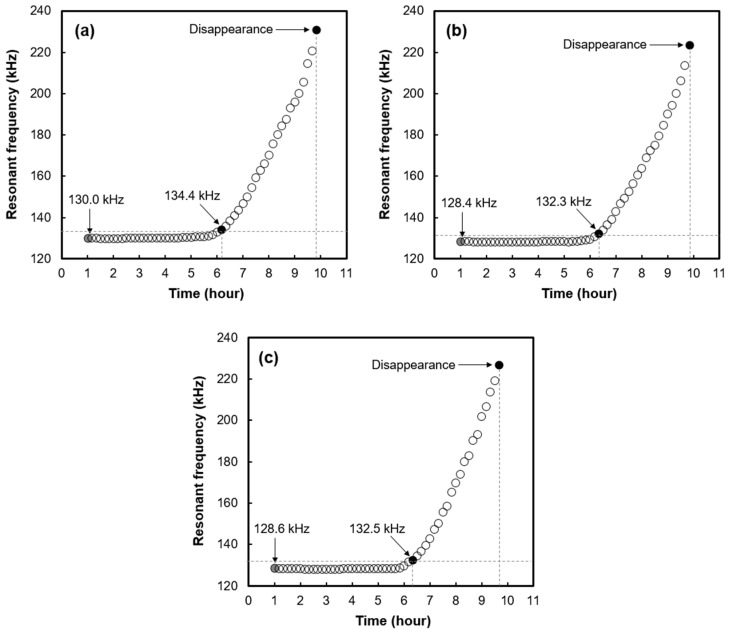
Resonant peak frequency of the EMI signal as a function of age: (**a**) C1, (**b**) C2, and (**c**) C3; concrete samples obtained from the same concrete batch.

Figure 4 shows the change in the resonant peak frequency of the EMI signal as a function of age. As shown in Figure 4, the EMI resonant frequency showed no significant change during the early stage of hydration. However, the EMI resonant frequency rapidly shifted to the high-frequency region at a specific time point in the hydration process.

In previous studies [13,14,15,16], it has been observed that when the PZT sensor is embedded in a cementitious material, there are no significant changes in the EMI resonant peak frequency during the early stages of hydration. However, at a specific time point in the hydration process, it has been observed that the EMI resonant peak rapidly shifts towards the high-frequency region and eventually disappears. When the stiffness of the material in contact with the PZT sensor increases, the EMI resonant peak frequency shifts towards the higher frequency region [13,21]. Cement paste exhibits a rapid increase in stiffness after the initial set during the hydration process [1]. Therefore, the time point at which the EMI resonant peak frequency starts to increase can be considered the initial setting time. In previous studies [13,14], the determination of the initial setting time was conducted using the tangent line method, which can be subjective and arbitrary. In this study, the initial setting time was defined as the point at which the EMI resonant peak frequency shifted to a higher frequency region by 3% or more compared to the initial measurement value of the resonant peak frequency. This 3% or more shift in the resonant frequency could be considered a substantial change that can be confidently discerned from measurement noise or other minor fluctuations.

The disappearance of the EMI resonant peak is attributed to the increased stiffness of material in contact with the PZT sensor, which dampens the free vibration of the PZT sensor [13,21]. As the cement paste gains rigidity, the coupling between the PZT sensor and the cement paste becomes stronger, resulting in the disappearance of the resonant peak [21]. Therefore, the time point at which the EMI resonant peak disappears can be considered the final setting time. In this study, a total of 10 min before the resonant peak disappeared was considered to be the final setting time.

Table 5 shows the initial and final setting time of concrete samples obtained from the same concrete batch using the EMI sensing technique with the embedded PZT sensor. The setting times of the concrete samples from the same concrete batch, as determined by the EMI sensing technique, did not show significant differences. The difference in the initial setting time between C1 and C2 was 10 min, and the difference in the final setting time between C1 and C3 was also 10 min. Considering the EMI signal measurement time interval of 10 min in the EMI sensing technique, it can be concluded that the observed difference of 10 min in the initial and final setting times falls within the measurement deviation range. It is important to note that the time gap between the initial and final setting times, as determined by the EMI sensing technique, showed remarkable similarity. The close similarity observed among the initial setting time, final setting time, and the time gap between the initial and final set, as measured by the EMI sensing technique, provides strong support for the reliability and accuracy of this technique in assessing concrete setting.

**Figure 5:**
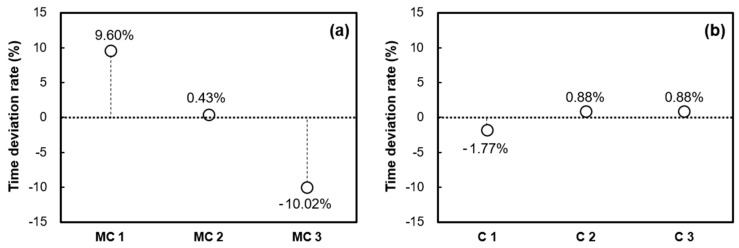
Time deviation rate of initial set: (**a**) penetration resistance test using mortar samples extracted from the same concrete batch and (**b**) EMI sensing technique using concrete samples obtained from the same concrete batch.

**Figure 6:**
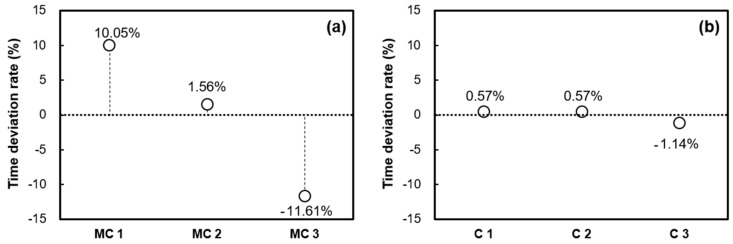
Time deviation rate of final set: (**a**) penetration resistance test using mortar samples extracted from the same concrete batch and (**b**) EMI sensing technique using concrete samples obtained from the same concrete batch.

Figure 5 and Figure 6 provide further insight into the time deviation rate for each sample, focusing on the average initial and final times. This assessment is carried out using both the penetration resistance test and the EMI sensing technique. Specifically, when the penetration resistance test was conducted using extracted mortar from concrete as a sample, it resulted in a time deviation rate of 10.02% for the initial setting time. In contrast, the EMI sensing technique, which employed concrete as a sample, showcased a notably lower time deviation rate of 1.77% for the initial setting time. When considering the final setting time, the penetration resistance test exhibited a time deviation rate of 11.61%, while the EMI sensing technique displayed a considerably reduced time deviation rate of 1.14%. These comparisons highlight the enhanced accuracy and precision of the EMI sensing technique in gauging concrete setting times compared to the conventional penetration resistance test.

The penetration resistance test, which uses mortar samples extracted from concrete, is not a direct method for measuring the setting time of concrete. As shown in the experimental results, the extraction of mortar from concrete through sieving can result in variations in the water/cement ratio and the cement/fine aggregate ratio, leading to differences in the setting time compared to pure mortar. In contrast, the EMI sensing technique offers a direct approach to assess the setting time of concrete. By embedding a PZT sensor directly into the concrete sample, the EMI sensing technique allows for the change in the stiffness of the concrete itself to be monitored during the hydration process. Through measuring and analyzing the variations in concrete stiffness using the PZT sensor, the EMI sensing technique provides an effective and accurate means of evaluating the setting time of concrete. This direct measurement approach provides valuable insights into the behavior of the concrete during the setting process.

## 4. Conclusions

This study investigated the evaluation of concrete setting times through two distinct approaches: the conventional penetration resistance test utilizing mortar extracted from concrete, and the electro-mechanical impedance (EMI) sensing technique employing directly embedded piezoelectric (PZT) sensors within the concrete.

The penetration resistance test showed limitations due to the procedure of extracting mortar from concrete using sieving. This extraction process has the potential to introduce variations in the ratios of water–cement and cement–fine aggregate, which in turn can result in disparities in setting times when compared to pure mortar samples. The experimental results highlighted that even for mortar extracted from the same concrete batch, the time deviation rate of the setting time exceeded 10%.

On the other hand, the EMI sensing technique offers a direct measurement approach that overcomes the limitations of the penetration resistance test, providing more reliable and comprehensive results for evaluating the setting time of concrete. This approach entailed the direct embedding of PZT sensors within concrete samples, enabling the real-time monitoring of stiffness changes during the hydration process. The EMI sensing technique harnessed the phenomenon of resonant peak frequency shifts, which responded to fluctuations in material stiffness. The experimental results revealed that when the curing time of concrete samples from the same batch was evaluated using the EMI sensing technique, the observed time deviation rate fell within the measurement deviation range, with a maximum value of 1.77%.

Therefore, the EMI sensing technique with an embedded PZT sensor presents a promising method for accurately evaluating the setting times of concrete. Further research and development in this area can contribute to the advancement of concrete quality control and construction practices.

## Figures and Tables

**Table 1:** Physical properties of aggregates.

Type	Specific Gravity	Maximum Size (mm)	Unit Weight (kg/m^3^)	Water Absorption (%)	Fineness Modulus
Fine aggregate	2.60	4.75	1564	1.05	2.63
Coarse aggregate	2.65	25.00	1569	0.9	6.49

**Table 2:** Mixture proportion of concrete.

W/C (%)	S/a (%)	Unit Weight (kg/m^3^)		
		Cement	Fine Aggregate	Coarse Aggregate
50	37.9	388	663	1088

**Table 3:** Specification of PZT sensor.

Frequency (kHz)	Resonant Resistance (Ω)	Capacity (pF)	Metal
3.5 ± 0.5	350	30,000 ± 30	Brass

**Table 4:** Setting times of mortar determined by ASTM C 403.

Designation		Initial Setting Time (min)	Final Setting Time (min)	Time Gap between the Initial Set and Final Set (min)
Mortar sample extracted from the same batch of concrete	MC 1	430	635	205
	MC 2	394	586	192
	MC 3	353	510	157
Pure mortar		373	576	203

**Table 5:** Setting times of concrete determined by EMI sensing technique.

Designation		Initial Setting Time (min)	Final Setting Time (min)	Time Gap between the Initial Set and Final Set (min)
Concrete sample from the same concrete batch	C 1	370	590	220
	C 2	380	590	210
	C 3	380	580	200

## Data Availability

Not applicable.
